# Supplementary material for: Whole-Genome Sequencing Reveals the Genomic Characteristics and Selection Signatures of Hainan Black Goat
Source: Genes (Basel). 2022 Aug 26;13(9):1539. doi: 10.3390/genes13091539 (PMC9498695; doi:10.3390/genes13091539)
Supplement: Supplementary file 1 [file genes-13-01539-s001.zip › genes-1856119-supplementary.pdf]

Supplementary Table S1. The origin of raw sequencing data.

Accession	BioProject	Breed	Accession	BioProject	Breed
SRR11744811	PRJNA631433	Longlin	SRR7476937	PRJNA479946	Dazu
SRR11744810	PRJNA631433	Longlin	SRR7476938	PRJNA479946	Dazu
SRR11744809	PRJNA631433	Longlin	SRR7476939	PRJNA479946	Dazu
SRR11744808	PRJNA631433	Longlin	SRR7476940	PRJNA479946	Dazu
SRR11744807	PRJNA631433	Longlin	SRR7476941	PRJNA479946	Dazu
SRR11744817	PRJNA631433	Longlin	SRR7476942	PRJNA479946	Dazu
SRR11744816	PRJNA631433	Longlin	SRR7476943	PRJNA479946	Dazu
SRR11744819	PRJNA631433	Longlin	SRR7476944	PRJNA479946	Dazu
SRR11744818	PRJNA631433	Longlin	SRR10083585	PRJNA560446	Jining
SRR11744815	PRJNA631433	Longlin	SRR10083586	PRJNA560446	Jining
SRR5803158	PRJNA631433	Longlin	SRR10083590	PRJNA560446	Jining
SRR5803161	PRJNA631433	Longlin	SRR10083598	PRJNA560446	Jining
SRR5803162	PRJNA631433	Longlin	SRR10083599	PRJNA560446	Jining
SRR5803163	PRJNA631433	Longlin	SRR10083608	PRJNA560446	Jining
SRR5803165	PRJNA631433	Longlin	SRR10083609	PRJNA560446	Jining
SRR6246091	PRJNA399234	Leizhou	SRR10083611	PRJNA560446	Jining
SRR6246084	PRJNA399234	Leizhou	SRR10083612	PRJNA560446	Jining
SRR6246085	PRJNA399234	Leizhou	SRR10083613	PRJNA560446	Jining
SRR6246086	PRJNA399234	Leizhou	ERR2309118	PRJEB25062	Boer
SRR6246087	PRJNA399234	Leizhou	ERR2309119	PRJEB25062	Boer
SRR15440572	PRJNA754269	Hainan	ERR2309120	PRJEB25062	Boer
SRR15440571	PRJNA754269	Hainan	ERR2309121	PRJEB25062	Boer
SRR15440564	PRJNA754269	Hainan	ERR2309122	PRJEB25062	Boer
SRR15440563	PRJNA754269	Hainan	ERR2309123	PRJEB25062	Boer
SRR15440562	PRJNA754269	Hainan	ERR2309124	PRJEB25062	Boer
SRR15440561	PRJNA754269	Hainan	ERR2309125	PRJEB25062	Boer
SRR15440560	PRJNA754269	Hainan	ERR2309126	PRJEB25062	Boer
SRR15440559	PRJNA754269	Hainan	ERR2309127	PRJEB25062	Boer
SRR15440558	PRJNA754269	Hainan	SRR4053642	PRJNA338022	Alashan
SRR15440557	PRJNA754269	Hainan	SRR4052479	PRJNA338022	Alashan
SRR15440570	PRJNA754269	Hainan	SRR4053576	PRJNA338022	Alashan
SRR15440569	PRJNA754269	Hainan	SRR4053547	PRJNA338022	Alashan
SRR15440568	PRJNA754269	Hainan	SRR4053519	PRJNA338022	Alashan
SRR15440567	PRJNA754269	Hainan	SRR4053460	PRJNA338022	Alashan
SRR15440566	PRJNA754269	Hainan	SRR4053410	PRJNA338022	Alashan
SRR15440565	PRJNA754269	Hainan	SRR4053363	PRJNA338022	Alashan
SRR7476927	PRJNA479946	Dazu	SRR4053244	PRJNA338022	Alashan
SRR7476929	PRJNA479946	Dazu	SRR4053213	PRJNA338022	Alashan
SRR7476931	PRJNA479946	Dazu	SRR4052677	PRJNA338022	Alashan
SRR7476932	PRJNA479946	Dazu	SRR4052649	PRJNA338022	Alashan
SRR7476933	PRJNA479946	Dazu	SRR4052601	PRJNA338022	Alashan
SRR7476934	PRJNA479946	Dazu	SRR4052590	PRJNA338022	Alashan
SRR7476935	PRJNA479946	Dazu	SRR4052525	PRJNA338022	Alashan
SRR7476936	PRJNA479946	Dazu			
